# Global Catastrophic Risk and the Drivers of Scientist Attitudes Towards Policy

**DOI:** 10.1007/s11948-022-00411-3

**Published:** 2022-10-28

**Authors:** Christopher Nathan, Keith Hyams

**Affiliations:** Politics and International Studies, University of Warwick, Coventry, UK

**Keywords:** Risk, Global catastrophe, Policy, Research culture, Responsible Research and Innovation

## Abstract

An anthropogenic global catastrophic risk is a human-induced risk that threatens sustained and wide-scale loss of life and damage to civilisation across the globe. In order to understand how new research on governance mechanisms for emerging technologies might assuage such risks, it is important to ask how perceptions, beliefs, and attitudes towards the governance of global catastrophic risk within the research community shape the conduct of potentially risky research. The aim of this study is to deepen our understanding of emerging technology research culture as it relates to global catastrophic risks, and to shed new light on how new research governance mechanisms might be developed. We analyse in-depth interviews with leading AI and biotech researchers both from universities and the private sector. We develop new insights in terms of four salient themes. First, ‘engineering mindset’, which highlights the premium placed by many interviewees on pursuing interesting research about the physical world for its own sake. Second, ‘self-government’, which looks at how self-regulation of technological development currently occurs. Third, ‘pure incentives’, focussing on how career and other incentives shapes research. Fourth, ‘norms and persuasion’, which examines the role of moral considerations in guiding the research choices of scientists. We end by considering the implications of these findings for future research on governance of anthropogenic global catastrophic risk.

## Introduction

The aim of this study is to deepen our understanding of research culture as it relates to global catastrophic risks. What are the perceptions of, beliefs about, and attitudes towards the governance of global catastrophic risk within the research community on emerging technology? The chances of a catastrophe caused by emerging technology could be small, but the consequences, if they eventuate, would be overwhelming, and the specific probabilities of their doing so are hard to know. A significant literature argues that such risks are due far greater attention than they currently receive, on several grounds including that such risks can be expected to be systematically neglected, that future generations deserve significant attention, and that unforeseen technological risks can eventuate very quickly when they arrive (Bostrom, 2013; Posner, 2004; for an overview see Ord, 2020). Moreover, given the number of lives at stake if humanity were to bring about its own extinction, philosophers have argued that even a small risk of extinction must be taken very seriously (Parfit, 1984). We suggest that understanding the cultures within research communities is an element of understanding how such risks can be assuaged and is therefore of great significance. Here, we present the results of a qualitative study aimed at developing this understanding.

For methodological reasons of focus, as discussed below, we limit ourselves to the fields of artificial intelligence and biotechnology. Grace et al. (2018) find, in a survey of experts, a median expectation of 2061 as the year at which artificial general intelligence (AGI) is expected to be able to accomplish any task more effectively than humans. The consequences of such a technology would be vast. Beyond this, it is difficult to say with credible certainty anything about the implications of AGI. A common refrain in the literature is the ‘alignment’ concern. This refers to the possibility that an artificial intelligence might come to have complex goals that do not reflect human values, but which the machine is able to pursue, given its abilities, despite human efforts to prevent it from doing so (Bostrom, 2014; Everitt et al., 2018; Hubinger et al., 2019; Russell, 2019). Aside from the alignment problem, the transformations that would accompany the creation of an advanced artificial intelligence might combine with other risks or structural weaknesses, and catastrophe may thus result. For instance, we might see the rise of AI nationalism or mercantilism (Dafoe, 2018), or vast effects on economic inequality (Korinek & Stiglitz, 2017), an accompanying increase in global conflict, leading to what has been referred to as a ‘boring apocalypse’, that is, a gradual decline of humanity caused by exposure of underlying vulnerabilities (Liu et al., 2018).

In the area of biotechnology, we focus on DNA synthesis, and the ‘gain of function’ controversy (DiEuliis et al., 2019). Regarding the former, it has been known for nearly two decades that it is possible to synthesise a full viral genome. In this time, synthesis technologies have developed and become cheaper to deploy, and a global industry now supplies DNA to order. As a recent report points out, ‘no governments currently require screening for DNA synthesis’ (World Economic Forum and Nuclear Threat Initiative, 2020), and a public concern has thereby arisen about malicious or negligent actors misusing pathogen DNA (Piper, 2020). The gain of function question relates to research in 2011 that involved adding functions to the H5N1 virus so that it became transmissible between ferrets. The ultimate goal of the work was to understand better how harmful viruses transmit between humans. Following reports in the popular press describing the deliberate creation of a doomsday virus, alongside concerns raised from within the scientific community, the publication of the research was halted, and a (now revoked) pause on the funding of all gain of function research was put in place in 2014. The issue precipitated a divide in the research community. Those running the experiments argued that their critics overstated the likelihood of lab escapes and ignored recent developments in biosecurity (Fouchier, 2015; c.f. Lipsitch, 2018).

Work on global catastrophic risk (GCR) in general expresses reason for a degree of scepticism about the idea that research that presents a possible danger will be easily reigned in (e.g., Ord, 2020; Bostrom et al, 2016; Critch & Krueger, 2020). Nonetheless, that GCR literature lacks concerted efforts to set out how researchers see their work and these issues playing out in their research communities. This study aims to fill the gap. The broad research questions guiding our approach were, first, what scope is there for improving governance of GCR arising from emerging technology by focussing on practices and assumptions within the scientific research community itself, rather than high level policy processes? Second, what are the obstacles to, and opportunities provided by, such an approach to GCR governance? And third, how does GCR governance at higher policy levels interact with actual practices and views within scientific research communities?

The themes discussed in the paper interact with work on scientist research culture more broadly (e.g. Moore, 2008; Mukerji, 2014; Wolfe, 2012) and with the literature on Responsible Research and Innovation (RRI) (Wiarda et al, 2021). Work on social theory and Responsible Research and Innovation informed by Beck (1992) argues for the necessity and inevitability of greater reflexivity of the scientist, given the increasing complexity of forecasting risks, seeing this as a ‘condition of contemporary modernity’ (Genus & Starling, 2018). In particular, one of the key issues to arise within the literature on RRI is the multi-layered nature of responsibility for implementing RRI (Fisher & Rip, 2013), and especially the distinction between role responsibility and collective responsibility (Grinbaum & Groves, 2013). Systemic constraints arising from political and economic background structures pose barriers to effective RRI measures, while at the individual level there are questions around how incentives and motivations can be aligned with RRI imperatives. Our research aims to show how, above and beyond the difficulties that arise within RRI implementation in general, the long-term, low-probability high-impact nature of GCR raises particular problems as a result of which there is a significant blind spot towards GCR issues within RRI practices. We discuss this further at the end of the paper.

The paper proceeds as follows. In the following section we outline the method that we adopted in selecting interviewees, conducting interviews, and analysing the results. Section three describes the results of our study, organising the findings into four themes which we argue best capture the strands of insight emerging from the research. Section four discusses the overall picture emerging from the results and the implications of our findings for GCR research and policymaking.

## Method

We approach these questions by sampling purposively from the scientific community, seeking a diverse group of participants who will provide an information-rich data set, with the goal of insight and understanding of the landscape of ideas and perceptions. For the purposes of striking a balance between obtaining a diverse data set, on one hand, and obtaining a data set that contains explicit and implicit connections, on the other, we limit the subjects to two areas: artificial general intelligence, and biotechnology relating to pathogens (on purposive sampling, see Patton, 2002). Such a focus is made with the goal of obtaining the most insight from the available research resources (see Robinson, 2014). Our method might inform or complement future studies including those relying on representative samples.

The study is based on a series of fifteen in-depth semi-structured interviews with researchers and technologists in commercial, university and civil society settings. More than a third of the respondents are currently at the top of their field, and half of the remainder are very senior. With its involvement of senior individuals, and its focus upon eliciting and probing perceptions, the study falls within the definition of a medium sized project, in Braun and Clarke’s (2013) terms (see also Malterud et al., 2016). The sample size was deemed sufficient in this context both because of the seniority of the interviewees and because of the nature of the qualitative research being undertaken, namely eliciting and probing perceptions. Interviewees were selected through three methods. First, we leveraged existing contacts and contacts of colleagues working in relevant research areas. Second, we used a snowballing technique to identify other potential interviewees from the initial set of contacts. Third, we identified and sought to remedy gaps and inequalities in the distribution of our coverage—in terms of level of seniority, area of research, geographical location, gender, and familiarity with policy processes—in order to ensure a diverse set of interviewees.

As it turned out, four fifths of participants were working in the U.S., with the remainder in EU countries. Approximately half worked on AI while the other half worked on biotech, with a gender split of two thirds male and one third female. The study does not aspire to statistical representativeness, and the sample is diverse in terms of seniority level, technology focus, and role. Further, it might be said that many scientists behave and see themselves foremost as a part of a global community focusing on their particular niche, and are best understood with respect to that niche. Nonetheless, areas for future study would be the questions of the extent to which research culture in the U.S. is distinct from European contexts and indeed from other important contexts, including that of China.

Interviews lasted between thirty and eighty minutes, and were professionally transcribed and pseudonymised. The topic guide covered five general areas: the interviewee’s background; their view of the specifics of global catastrophic risks; their view on the efficacy of the governance of these risks; what, if anything, might be done to improve such governance; and the locus or impetus for such improvements. As the subjects are theoretically mature, and indeed several have published work setting out their views, the interview form is especially appropriate, in providing a way to understand respondents’ underlying assumptions, and to actively translate terms and concepts between respondents. Several provided, unprompted, an article or document that they had authored that seemed to them to be relevant to the subject. In such cases, this would provide some of the impetus for the discussion. In other cases, the interviewer became familiar in broad terms with the subject’s work.

In addressing the research questions, our approach both to the structure of interviews and to the subsequent analysis took place through what Braun and Clarke (2019; see also Braun & Clarke, 2006) now call ‘reflexive thematic analysis’, whereby codes are permitted to evolve throughout the study. More specifically, once the research design was established and institutional ethical approval was in place, the process began with interviews being recorded and professionally transcribed. Transcriptions were read through and given an initial coding, codes were examined for overlaps or hierarchies, the transcripts were re-read in the light of this, allowing for the development of new codes. Coding took place in a reflexive manner, seeking to find signifiers for patterns of meaning across the data. Significant codes were then promoted or amalgamated into *themes*, these being central insights or concepts within the text. An example of the process is the way in which the code ‘scientist neutrality’ arose and developed. This denotes an idea that was expressed using different languages across the sample set, and accordingly, as the analysis progressed, it was placed hierarchically above other codes referring to scientists as ‘engineers’ and a ‘creativity imperative’. The reflexive coding approach allows and indeed embraces the view that ‘new meanings are always (theoretically) possible’ (Braun & Clarke, 2021). While allowing the productive element of the researcher in constructing themes, the analysis was triangulated by the second author, working on the draft of the analysis, checking its validity and challenging its coherence.

The four themes that emerged from our interviews, guided both by our research questions and by the reflexive thematic analysis approach, were: ‘Engineering mindset’, ‘Self-government’, ‘Pure incentives’, and ‘Norms and persuasion’. The engineering mindset theme captures the premium placed by many interviewees on pursuing interesting research for its own sake. A sub-theme of the engineering mindset is ‘Exceptionalism’. This refers to a pattern whereby where researchers do put themselves forward as taking a view on matters of ethics or policy, it is offered as a way of setting themselves apart from other researchers. The self-government theme looks at the ways in which self-government of technological development currently occurs, or fails to occur, and its potential for development. Pure incentives focuses on the strong effect that career and other incentives has on shaping what sort of scientific research happens, suggesting that some types of incentive can work to deter the development of technology that can exacerbate GCR, while other types of incentive can inadvertently encourage it. Finally, norms and persuasion examines the role of moral considerations in guiding the research choices of scientists. Together, our findings on each of these themes paint a broad overview of the existing state of play of GCR governance as experienced by members of the scientific research community itself, as well as thoughts on how GCR governance might be improved.

## Results

### Theme 1. Engineering Mindset

In talking to scientists and technologists about research culture and the governance of science, it is difficult not to be struck by the primacy of what might be called the *engineering mindset*. This is an overriding focus on understanding a physical phenomenon, on building new theories or models of it, on showing how something functions and on putting ideas into practice. It constitutes a strong curiosity about some aspect of the world. One AI researcher says: ‘I’m certainly a lot happier and at ease if I’m just focusing on understanding and building stuff’ (S1).^1^ ‘Understanding and building stuff’ is contrasted, here, with arguing about or designing policy for governing risks from science and technology. We can see from this that the curiosity associated with the engineering mindset can crowd out curiosity about other aspects of the world, namely, those associated with norms and policy. Another senior AI researcher states, ‘I’m much more interested in building the technology.’ As the conversation on policy continues, he later says, ‘I would rather live in a world where I could just not think about that stuff at all because it’s a combination of finding it…boring and distressing.’ (S2) We see here how the engineering mindset can be self-aware: it need not deny the possibility that there is value in researchers placing some focus upon the broader implications of their work; rather, it expresses a strong draw towards technical and scientific discovery.

The following is submitted by a senior AI researcher early in an interview, prompted only by a spiel about the study and a question about the interviewee’s background:

‘I consider myself more of a scientist… For people like me, actually we don’t really care too much about the application, it’s more like we are curious, … we really want to know what thinking is about.’ (S3).

In this case the curiosity imperative of the self-identified ‘scientist’ resides at an abstract level, and doesn’t extend to application (implicitly, a matter for engineers and other practitioners), let alone the proper rules governing the application.

The crowding out of concerns with policy by the engineering mindset is also illustrated by the view of a microbiologist who now works directly on policy: ‘I think most scientists kind of keep their heads down and just do the work… I’m sure most scientists don’t have an opinion on it.’ (S4) An experienced epidemiologist states:

‘Governance is an issue that I think some people have a passion for and an intuition for. And for me, I think it’s a thing that needs to happen. I don’t really understand how it comes about or why it fails, but I recognise the importance, just I’m not a big mover in that circle.’ (S5).

Whether as active aversion or mere disinterest, what we see here is an implicit driving idea that interest in matters of governance is a *preference*: as a scientist, one can coherently choose not to engage. And further, once this space is established, we see how the engineering mindset can act to drive out concerns with policy.

The primacy of the engineering mindset over concern with broader governance and social impact is often alluded to as an aspect of the worldview of interviewees’ colleagues. A leading biologist states, ‘some of my colleagues…generally don't want to be governed.’ (S6) Another researcher states, ‘most people doing technical AI research, direct research on propelling the capabilities of AI forward, would I’d say largely be against any form of governance of their research.’ (S7) These comments arise as part of general discussion of how to govern the field. This view is in some cases expressed in terms of *exasperation* or *regret*. In their failure to consider the implications of AGI, one states, ‘there's a lot of growing up in general that I think scientists have to do.’(S7) The interviewee takes the view that the ‘joy and pleasure of discovery’ is so powerful that some reason to continue any avenue of research will be found regardless of the dangers. Similar frustration arises elsewhere: ‘I don’t think the people who are working on the horsepox thing had, even though I tried to warn them,…any idea that it was going to be as controversial as it was.’ (S4)^2^ In accordance with the exasperation, interviewees would present themselves as exceptions to the rule. Call this *exceptionalism*. A wide range of specific policies and governance strategies were proposed, discussed, and criticised in a lively and well-articulated manner, including:

- Reforms to publication norms in science journals relating to potentially dangerous information;
- Encrypted centralised or distributed DNA screening mechanisms;
- The recent moratorium on ‘Gain of Function’ research;
- Certification for DNA synthesis companies;
- Mechanisms for recognition of legal personhood of advanced artificial intelligence;
- The expansion of GDPR so that audit trails are required for large transfers of data.

Furthermore, discussions of the possible threats to humanity on the horizon would extend beyond respondents’ own specific expertise, and often would focus on an area adjacent to their own. This reflects an interest in policy issues that runs beyond scientific interest and expertise in a particular phenomenon. One researcher, having seen and experienced the way that governance of DNA synthesis developed, and internalised the norms around it, is ‘amazed’ by the absence of such norms in the cognate area of de novo protein design (S10). Talking about AGI developers, one computer scientist states: ‘computer scientists are… aware that there’s somebody out there who’s going to have to build institutions and governments and laws… to control this thing that we’re building, but they’re just thinking that it’s somebody else’s job.’ (S1) Another interviewee expresses doubt that those developing new weapons will be able properly to identify and contain the danger of information about them spreading (S12).^3^

A narrative that emerges is of the individual scientist who is motivated centrally by the engineering mindset in their own work, and who in other modes takes a view on governance matters, though does so in a setting in which others are perceived not to take any such view, and in way that focuses on adjacent fields.^4^ We might call the combined resulting practices ‘Collingridge misalignment’. Collingridge (1980) urged that by the time that technologies have been developed, lock-in effects can render it too late to reform them. Those working at early technology readiness levels have, therefore, more influence than they first might seem to have. Collingridge misalignment involves (a) those scientists with immediate power over some technology’s direction disregarding the value of the influence that they have, and (b) scientists in adjacent areas taking an interest in the technology’s governance relating to GCR, while lacking immediate influence over its direction. In general, according to this theme, the points at which GCR from emerging technologies tend to be engaged at an early Technology Readiness Level do not match the expertise of the ‘non-depoliticised’ on that specific issue—that is, there is a tendency towards either *adjacent critique*, or a disregard of Collingridge considerations and a focus on understanding a physical phenomenon.

### Theme 2. Self-government

Respondents display an instinctive understanding of the interaction between top-down regulations and the practices of science, and of the significance of the latter with regard to governance of GCR, even if it is not put in terms of ‘governance’. A representative statement of such understanding of the interaction between top-down regulations and the practices of science comes from an AI expert, emphasising the tension between putting control in the hands with those with expertise, and putting ‘the foxes in charge of the chicken coop.’ (S13).

When asked about governance, some paint a relative free-for-all. In the AI world, as an element of ICT, ‘self-governance is the only thing that seems to be happening.’ (S7) In biotech ‘our problem is not that we desperately want to self-govern, it's that we can't get anyone else to govern us.’ (S6) The view is also expressed that ‘it’s not just that [self-government is] the best thing we could do, it’s really our only option…’. (S4) That is, the expertise necessary to understand the policy issues is at the cutting edge, and so it is inevitable that good governance will involve those doing the research taking an active part in governing.

In the area of gene and DNA synthesis, several respondents referred to the International Consortium for Gene Synthesis in discussing this topic. The shortcomings of this body are noted: in particular, that it is largely voluntary, and covers only around 80% of orders. Nonetheless, it is well-regarded and pointed to as a model that might be further developed. One scientist who was involved in setting up the consortium referred to the opposition he faced when setting it up, having to ‘stare down’ those who argued that a synthesis company would have no responsibility in a scenario in which it sold smallpox to a client who released it (S6).

Respondents also note the way that self-governance arises from the private sector, and indeed how the private sector takes the lead in providing governance models. One, whose professional role includes both research and policy advocacy, sees this work as ‘serving as a technical resource for the US and other governments’, where ‘regulatory frameworks will lag behind’ because of the fast-moving nature of the technology (in this case, synthetic biology). (S10). Nonetheless, the kind of agreement that arose from the Asilomar conference in 1975, which is sometimes presented as a good example of self-governance by technologists,^5^ tends to be distinguished by interviewees as unlikely to arise today. One notes that self-regulation of the gene-synthesis industry is driven by the private sector, whereas Asilomar grew from academics. Although, ‘the core question is still the same which is how do we ensure that the research and technology we’re working on is used responsibly and that we have a role in that’. (S10) Moreover, another takes the stance that the necessary attitude on self-regulation is not currently in place: ‘If anything, I think the biology community has walked away with the notion that there was nothing to worry about’, since recombinant DNA has turned out to be quite safe; genes are successfully and safely grown in laboratories in *E. Coli*. (S8).

There is also a significant absence in this theme. On the idea that a technology might be governed by the people that are subject to it, that is, all of the stakeholders, one respondent replies, ‘that's an interesting question. I struggle to think about how that could happen technically or politically. But certainly, I would be in favour of that.’ (S7) The term ‘stakeholders’ or any equivalent does not naturally come up in conversations. Self-government refers to scientists taking part in the process, rather than all of those affected by it. The exception is a scientist who works for the European Commission, who actively advocates and works in foresight analysis. (S9).

In summary, the instinctive understanding of the way that governance must work, as an interaction between the experts and those with executive power, is manifest, and displayed in the numerous ways in which power is exercised by those with expertise. The point extends to the private sector and to voluntary agreements, but not to more dramatic democratic proposals.

### Theme 3. Pure Incentives

In their self-understanding of research culture with regards to governance issues, there is a focus on incentives, and especially economic incentives, as the driver of research culture. That is, economic incentives are seen as the primary explanation for the current state of the culture, and as the natural first step for possible reforms. A senior microbiologist argues that one reason that the rules on research ethics are respected is the ‘altruistic’ reason that ‘we're all in this together.’ He continues: ‘Whether we do research on these viruses or not, we're all part of the human population that would be affected by any catastrophic event or accidental development of a pathogen, so we are therefore sensitive to the concerns of everyone’ (S16).

That this reason is described as ‘altruistic’ is indicative of the focus on incentives. Many will take the view that an act is not altruistic if it is in the actor’s own interests; a conceptual universe that takes the contrary view is perhaps one that has less room for non-self-interested behaviour. A similar argument that ‘everyone is in the same boat’ arises in another interview, without the altruism moniker, concerning DNA synthesis, whereby it is urged that it would be economically ruinous for a company to be seen to be responsible for the sale of DNA that was ultimately used to release a lethal and infectious virus. (S10) On this narrative, there are many confluences of the economic self-interest of those at key points in the research and development of emerging technologies, and of the nature of the good governance of those technologies with regard to possible catastrophe.

Negative judgments of existing research culture as it relates to governance are also put through the lens of economic incentives. Several respondents note that the best remuneration in their areas is for technical work, and so the best problem-solvers are drawn to doing this work, rather than applying their minds to solving the political problems. One describes the ‘brain drain’ from Washington DC to California of those with technical expertise who take some interest in policy issues, but then turn back to research in view of the salary that they can command. (S7).

On the whole, academic incentives are considered to be well-aligned with governance. As one urges, ‘you need funding to do research and if you don't respect the rules, you won't get the funding.’ (S16) Further evidence that academic structures are looked upon in a broadly positive way is an exchange in which a senior academic who also has a role in the private sector on AI development is pressed on what might be done to improve the technology now, so that it might be more attuned to these possible risks at this stage. The respondent replies, after some hesitation, ‘you know…you could regulate big tech.’ (S2) It is notable, that the more forceful policy landed upon is considered to be the regulation of the private sector, rather than a restructuring of the research phase, that is, of academic incentives. Another respondent similarly urges delegation to existing academic structures. In this view, the WHO guides a research agenda with input from experts from a global community of experts, and sets out guidelines for how this research should be conducted. (S15) A source of failure of regulation, from this perspective, is insufficient delegation to the scientific community.

An exception to the tendency not directly to criticise academic incentive structures comes in the following statement of the problem of information hazards (see also Bostrom, 2011):

‘if you try to warn the world that there is a horrifically destructive, highly accessible, new technology here, people will want to explore it. This is what academics do… And the more attention it receives, the greater the information hazard, the more complete a picture comes together of how to actually go about building it.’ (S12).

The concept of information hazards arises elsewhere in discussions. Even then, the above described view is not mainstream. Another respondent emphasises the inevitability of flows of information, where projects are worked on by multiple institutions and are subject to scrutiny by reviewers and conference audiences, and also expresses trust in the research community properly to balance the possible benefits and dangers of a research project. A further respondent accepts that the publication of the horsepox genome was an ‘eye opening moment for a lots of people in synthetic biology around thinking through what you should and shouldn’t publish,’ and dates the popularisation of the term ‘infohazard’ within the synthetic biology community to that moment, but he also expresses scepticism about efforts to change the existing publication system, emphasising practical problems with possible reforms, such as the danger of creating an ‘economic incentive for publication venues who don’t care or care less’ about information hazards, as well as the problem of editorial disagreement about whether some particular piece of information is hazardous, leading to ‘non-uniformity’ in policy across journals.

In general, then, there is a focus on incentives, and especially economic incentives, as the driver of research culture. While the views of the structure of these incentives in the private sector is mixed, the views of these in academia are more positive.

### Theme 4. Norms and Persuasion

There is a contrast between incentive-based and what might be called norm-based reasons for research culture. The following exchanges arises after the interviewee, a senior and successful biologist, had emphasised his attitude of finding ways to improve governance through identifying ways in which good governance also happens to be in the best interests of the governed:

CN: You must have spent a certain amount of time making your case rationally,…rather than just offering win–win situations…

S2: I don't really consider making a case for rationality or a rational case… I do explain what I'm doing. I like to be transparent, but I'm not trying to convince anybody.

CN: I suppose where I was going is that you, yourself, became convinced in some way or another through your own experience…

S2: Yeah, I guess that's true… I think I understand what goes on in other people's minds better than I understand what goes on in my own mind. (Laughs). (S6).

The simpler way—the low-hanging fruit—to reform is to find win–win situations. There is also awareness that, norms, fashions in thinking, and persuasion play a role. In this particular quote, the awareness is reluctantly admitted to: the interviewee finds reform success in working at the level of people’s straightforward incentives, and instinctively shies away from going beyond this realm. The awareness might be suppressed for the sake of focus on the lower hanging fruit. Still, there are several ways in which research culture is explained in terms that go beyond pure self-interest. Some instances of this are the idea that culture would change quickly in the event of a prominent disaster, such as 9/11 or COVID-19, as moments in which reform might occur.

Academic fashions also play a role. In a discussion on governance of DNA synthesis, an interviewee argues that we would benefit greatly from being able to determine from ‘primary sequence alone what the potential function of something might be’, but that such research is not currently given priority. When asked why, a number of research-cultural reasons were given: that funders prefer ‘sexier’ research that produces, for example, new kinds of material; that the work of doing such research may be ‘very boring’; that taking on long-unsolved problems can be off-putting, since there will tend to be a body of work holding that the problem is unsolvable. (S10) A further aspect of academic fashions is *intradisciplinary boundaries*. Examples of this arose in the discussion of ‘adjacent critique’ in the discussion of exceptionalism above, where respondents would find dysfunction in cognate research areas to their own. Furthermore, several respondents allude to the separation of the ‘AI safety’ community from AI researchers, with Stuart Russell as a prominent exception. One cites the ‘very interesting’ work coming from the Machine Intelligence Research Institute as carried by people who are ‘not AI researchers, most of them’, urging, further, that only a ‘small minority’ take the arguments for looking at misaligned AI seriously. (S2).

A small minority of respondents alluded to Effective Altruism (EA) as an aspect of understanding research culture^6^: not, of course, as a mainstream matter, since EA itself is not mainstream, but nonetheless as an independent force. One states, ‘a lot of Effective Altruism people or rationality adjacent people are the ones that manage to really stick with this topic [of governance] because they’re trying to be very organised with their priorities or their goals or values.’ (S1) Another, who actively works to reform academic structures in the light of concerns about excessive risk from emerging technology states, ‘I don’t identify as an effective altruist myself. But virtually, any effective altruist in the world would consider me aligned with their causes and interest.’ (S12) These statements suggest the form of frameworks that motivate scientists to adopt norms and attitudes about governance in general.

We have noted the reactive nature of policymaking; academic fashions regarding what counts as exciting research; cultural boundaries within disciplines placing work on making technology safe separately from making the technology itself; and the base level philosophical motivations of the researcher. Taken together, we see an understanding of scientist motivation that goes beyond the short term incentive and draws upon a wide variety of norms and practice, touching on the way that the structure of change in institutional context and individual outlooks plays a key role in understanding scientist attitude toward GCR.

## Discussion

We set out to identify perceptions, attitudes and beliefs with regard to GCR governance. It might be noted that discussions would turn to governance and ethics issues in general. GCR governance is, indeed, bound up with general governance. Scientists would often, as it were, switch into ethics and politics mode, and the mode includes a wide range of questions. A number of the themes set out above are not matters that arise exclusively with regard to GCR. They arise in discussions of governance more broadly, including those referring to subcatastrophic or moral risks, such as data bias, economic transformation, imposition of risks on particular communities, and bioethical matters (Owen et al., 2012). Nevertheless, our findings suggest that the nature of GCRs—in particular their long-termist nature, and the low-probability high-impact nature of the risks they pose—exacerbates these problems and raises new difficulties. A meta-theme of this study, then, is the boundedness of GCR governance with other ethical and governance matters within research culture. The boundedness is not always played out in real life. For example, frameworks such as RRI and ELSI do not cover GCR, which tends to be covered as an aspect of security, where it is covered at all. There is potential for greater integration here.

However, there is at least one way in which the themes described play a distinct and forceful role in discussions of GCR governance, as distinct from governance in general. This is due to the well-documented psychological confusion that accompanies high-impact, low-probability risks (Yudkowsky, 2008). GCR governance thereby has the character of an issue that is either easily dismissed, or the domain of a band of obsessives. The engineering mindset heightens this effect, providing the impetus and culturally accepted reasons and norms for disregarding the matter, even where sub-catastrophic risks are given space for discussion. This construction provides some explanation for what we described as Collingridge misalignment: where taking an interest is optional, one might expect that pattern of interest to be more erratic. In the case of GCR there are thereby two separate forces (the psychology of risk, and the engineering mindset) that drive a mismatch at the early development stage of technology between interest in policy and social issues and the ability to influence them.^7^

There are broadly two ways in which one might interpret what we have described as the frequent *exceptionalism* among respondents. First, it may be that others are incorrectly perceived, and that scientists care more about policy than they care to admit to one another. Second, it may be that others are correctly perceived, and a selection bias of this study gives voice to those willing to take a view. We can observe, either way, the narrative of the isolated opinion-holder. We would caution against generalising either possible interpretation without further study. What we provide here is the structure of a narrative that might be explored further and used as a framework for understanding.

Nonetheless, the theme here chimes with a number of other studies. Some describe a ‘technoscientific viewpoint’ emerging from interviews with science policymakers, a perspective that involves subjecting and apparently reducing ideological or value choices to complex technical decisions (Macnaghten & Chilvers, 2014; Smallman, 2020; Wynne, 1993, 2001).^8^ From a different angle, Smith-Doerr and Vardi (2015) describe the process of ‘purposive decoupling’, by which practicing scientists distance themselves from ethics rules and instruction, often through the use of humour. Similarly, in philosophy of science, there is sometimes an assumption that scientists, when they practice science, take themselves—perhaps mistakenly—to be doing something that is value-free (e.g., Douglas, 2009).^9^ Furthermore, within the literature on Responsible Research and Innovation, there is a growing body of research that identifies barriers to responsible innovation, including cultural barriers such as a tendency to see ethical matters as a box-ticking exercise, and provides reflection on what conditions would need to be in order to create a more reflexive scientific community, such as the ‘projectification’ of academic research (Felt, 2017; Wittrock et al., 2021).

The theme of the engineering mindset is a positive one. In contrast, ideas about value-freeness, indifference and aversion, purposive decoupling, non-reflexiveness, and the technoscientific viewpoint, are negative: they are defined by an absence of something that, implicitly, ought to be present. The engineering mindset describes a positive interest of scientists, who consequently may see political issues as frustrating or boring because their realms are prone to irrationality, or because they are seen as more difficult to progress. Behind the mindset is the motivation to be able to make progress in understanding the world, in what more than one interviewee describes as the ‘beauty’ of the phenomenon that they work on. Curiosity is reinforced by feedback, and feedback comes more easily for scientists in doing science.

For those interested in how to better govern potential catastrophic risk from emerging technology, an area for closer examination, then, is the way that scientists working in emerging technology do become drawn in to reflection upon the long term and social implications of their work, even where their professional role does not demand this: in this study, the effective altruism framework was noted. We might ask what it is about this framework that brings reflection in a way that lacks ‘purposive decoupling’, and how whatever this is can be broadened.

The study reveals insights about scientist perceptions of risks and policy around catastrophic risk. Understanding perceptions of a phenomenon, of course, is not identical to understanding a phenomenon itself. The former is an element of and a route into the latter. Were there a deeper understanding throughout the world of emerging technology of how, for example, scientific culture change can occur, then—given the instinctive understanding of self-governance that we identified—it might be easier to change it. Thus, the absence of RRI and ELSI from conversations about culture change is significant. It (a) further confirms that those mechanisms do not take on board, and indeed may be positively in tension with, GCR governance, and (b) indicates that a natural way to improve research culture and soft governance of GCR, namely, to incorporate such matters in to RRI/ELSI, is not within the horizons of scientists, and that, further, those agendas are seen as imposed from the outside, as indicated by, e.g., Hurlbut (2015). This theme arises in other research on RRI. There is a growing body of research that identifies barriers to responsible innovation, including cultural barriers such as a tendency to see ethical matters as a box-ticking exercise, and provides reflection on what conditions would be needed in order to create a more reflexive scientific community, such a diminution of the ‘projectification’ of academic research (Felt, 2017; Wittrock et al., 2021). The focus on incentive structures similarly indicates a sense among scientists of being subject to outside rule. Furthermore, one might surmise, even if it is not the dynamic in every case, the identified inclination to focus upon incentives as drivers of culture change has the effect of placing responsibility for practices upon external factors and not within the scientific community. It is thereby a route to non-reflexivity.

The overall picture arising from this discussion is as follows. The science research community has an important role to play in ensuring effective mitigation of global catastrophic risks arising from technological advances. Of particular concern to those seeking to improve governance of GCR ought to be the curiosity-driven trend to ‘follow the science’ regardless of potential consequences, coupled with the fact that Collingridge misalignment means that scientists themselves—rather than anyone external to the research community—are often best placed to identify likely risks on the horizon and potential lock in effects. Scientists have some degree of aversion or indifference towards considering policy issues. When they do not, they consider themselves to be doing it somewhat against a professional backdrop of indifference. Respondents display an instinctive understanding of the interaction between top-down regulations and the practices of science, and of the significance of the latter with regard to governance. The points at which GCR from emerging technology tend to be engaged at an early Technology Readiness Level do not match the expertise of the ‘non-depoliticised’ on that specific issue; they tend to be adjacent. Others seem to disregard the lock-in issue altogether. In understanding research culture with regards to governance issues, there is a focus on economic incentives as the driver of research culture. Other factors that arise are the reactivity of governance, academic fashions, and rational/moral persuasion.

Taken together, these themes suggest that promising directions for future governance include incorporating GCR in to existing incentivised norm structures (such as those codified and endorsed by funding bodies); promoting or better understanding what is attractive about the effective altruism framework; finding ways to reinvigorate further of the role of the public-facing scientist; popularising the Collingridge dilemma, especially as it applied to emerging technology and GCR, and placing under greater scrutiny the academic incentives in this regard; and of course, addressing more broadly the incentives towards short-termism in technology development. Further, it would be worth examining further the process of switching between technical and ethical modes, including examining the way that researchers respond to evidence about the way their reflection functions.

## Footnotes

1 Study subjects are identified with code numbers in brackets, preceded by an ‘S’.

2 A couple of respondents also indicate a generational divide in this regard, noting a greater concern among younger researchers with possible catastrophes from emerging technology.

3 There are exceptions, as one would expect. For example, a researcher working on artificial life expresses direct concern with the problem of avoiding misaligned AI. (S7).

4 We examine the coherence of the implications of this theme further in the Discussion section below, including the apparent tension between, on one hand, researchers wanting to focus on the science and ignore the governance, and, on the other hand, the frequent presence of exceptionalism.

5 At this conference, scientists self-limited research on recombinant DNA. The conference gives its name to a 2017 set of principles for governing AI.

6 Effective altruism is a movement encouraging people to do as much good as they can, using rational, evidence-based processes. See MacAskill 2015. It has recently tended to endorse the view that global catastrophic risks should be taken more seriously, and that misaligned artificial general intelligence is a real, if low probability, danger.

7 A further question for future research concerns the roots of the engineering mindset itself: is it an inevitable consequence of the specialisation that is necessary for technological progress here and now, or is it a more contingent result of the way that incentive structures (for example) are currently arranged?

8 See also Smallman’s (2018) description of an ‘elite sociotechnical imaginary of science to the rescue’.

9 But c.f. Robinson et al. (2016); Steel et al. (2018).
